# Contiguous Quaternary Carbons: A Selection of Total Syntheses

**DOI:** 10.3390/molecules25173841

**Published:** 2020-08-24

**Authors:** Alina Eggert, Christoph Etling, Dennis Lübken, Marius Saxarra, Markus Kalesse

**Affiliations:** 1 Institut für Organische Chemie, Gottfried Wilhelm Leibniz University Hannover und Biomolekulares Wirkstoffzentrum (BMWZ), Schneiderberg 1B, D-30167 Hannover, Germany; alina.eggert@oci.uni-hannover.de (A.E.); christoph.etling@oci.uni-hannover.de (C.E.); dennis.luebken@oci.uni-hannover.de (D.L.); marius.saxarra@oci.uni-hannover.de (M.S.); 2 Helmholtz Zentrum für Infektionsforschung(HZI), Inhoffenstraße 7, D-38124 Braunschweig, Germany

**Keywords:** canataxpropellane, taxane diterpene, waihoensene, *ortho*-photo-cycloaddition, natural products, total synthesis

## Abstract

Contiguous quaternary carbons in terpene natural products remain a major challenge in total synthesis. Synthetic strategies to overcome this challenge will be a pivotal prerequisite to the medicinal application of natural products and their analogs or derivatives. In this review, we cover syntheses of natural products that exhibit a dense assembly of quaternary carbons and whose syntheses were uncompleted until recently. While discussing their syntheses, we not only cover the most recent total syntheses but also provide an update on the status quo of modern syntheses of complex natural products. Herein, we review (±)-canataxpropellane, (+)-waihoensene, (–)-illisimonin A and (±)-11-*O*-debenzoyltashironin as prominent examples of natural products bearing contiguous quaternary carbons.

## 1. Introduction

In 2016, the groups of Overman and Hanessian published a well-arranged review about synthetic strategies for natural products bearing contiguous stereogenic quaternary carbons [1]. By focusing on already completed challenges in natural product synthesis, they adverted to still unsolved synthetic problems [1]. Within this review, taxane-derived propellane skeletons and terpenes, as well as terpenoids bearing contiguous asymmetric quaternary carbons, are mentioned to be one of the most exciting future challenges. In 2020, Gaich and coworkers published the first total synthesis of canataxpropellane (**4**) [2,3], a previously highlighted future challenge. Waihoensene (**23**) was successfully synthesized by Lee and coworkers in 2017 [4]. Three years later, syntheses of waihoensene (**23**) by the groups of Yang and Snyder followed [5,6]. Finally, a total synthesis of musabalbisiane A, mentioned in Hanessian and Overman’s review, is still not published [1]. Furthermore, this review covers a recent synthesis of (±)-11-*O*-debenzoyltashironin (**55**) by Wang and coworkers [7] and the first synthesis of illisimonin A (**72**) published by Rychnovsky and Burns [8].

## 2. Synthesis

### 2.1. Gaich’s Synthesis of Canataxpropellane

The Gaich group succeeded in the first total synthesis of a complex taxane diterpene, canataxpropellane (**1**).

**Figure 1:**
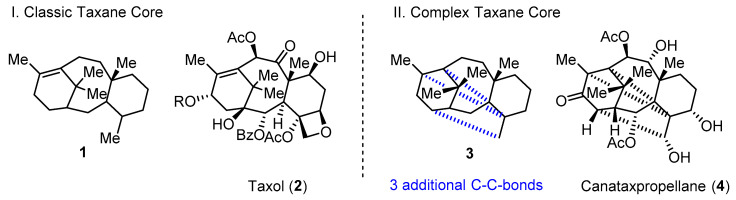
Comparison of the carbon backbone of taxol (**2**) and canataxpropellane (**4**).

The canataxpropellane (**4**) features three additional bonds (highlighted in blue) compared to the typical taxane core 1. Two of these additional bonds generate four quaternary stereocenters, and the third bond connects a methyl group to a former secondary carbon (Figure 1).

**Figure 2:**
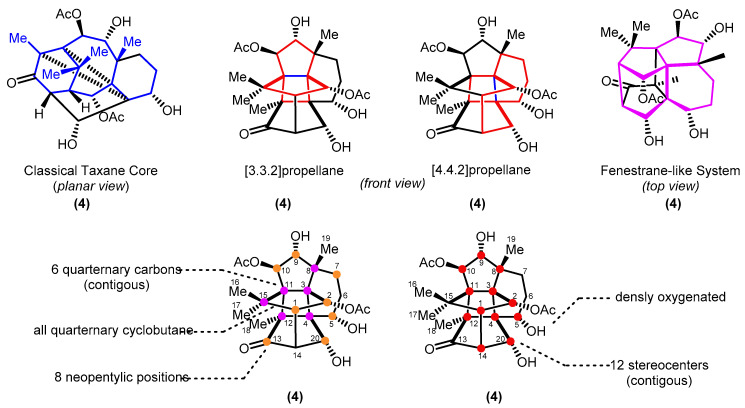
Different representations of canataxpropellane (**4**).

Looking from different orientations at the cantaxpropellane (**4**), one can identify a [3.3.2] or [4.4.2] propellane motif by looking from the front. Additionally, the top view visualizes a fenestrane-like framework. Overall, it features 12 stereogenic centers, of which 6 are contiguous quaternary carbons, and an all quaternary cyclobutane. This leads to eight neopentylic positions aside from these quaternary centers (Figure 2).

**Figure 3:**
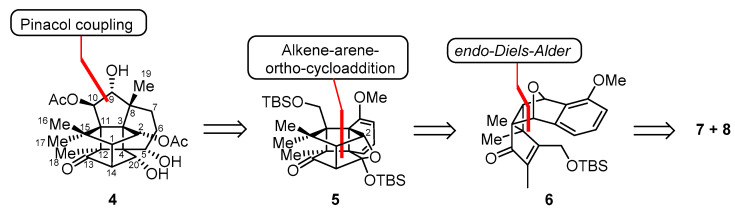
Structure of canataxpropellane (**4**) and Gaich’s retrosynthetic analysis [2].

The key steps in this synthesis are an *endo*-selective Diels–Alder reaction of an alkene-arene-*ortho*-photo-cycloaddition. Starting from inexpensive materials, they managed to obtain the final canataxpropellane (**4**) after 29 steps, starting from commercially available building blocks (Figure 3).

**Scheme 1:**
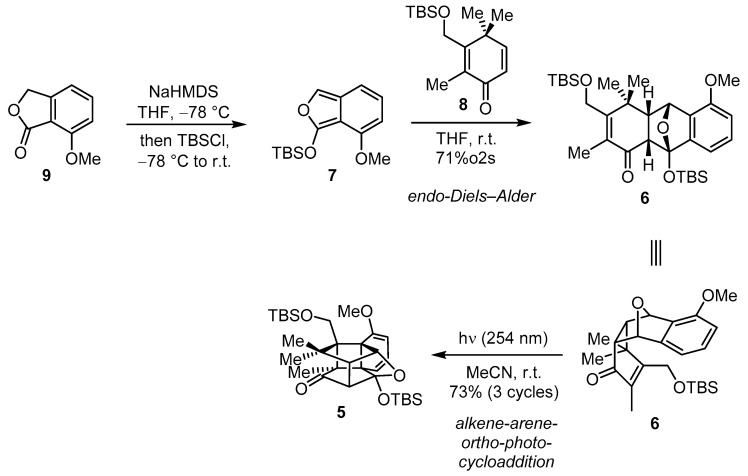
Gaich’s synthesis of propellane core **6** by alkene-arene-*ortho*-photo-cycloaddition towards the synthesis of canataxpropellane (**4**) [2]. TBS = *t*-butyldimethylsilyl.

Starting from lactone **9**, they generated silyl enol ether **7** for the envisioned *endo*-selective Diels–Alder cycloaddition as the requisite precursor for the alkene-arene-*ortho*-photo-cycloaddition (Scheme 1). The dearomatizing intramolecular [2+2]-photocycloaddition constructed the [4.4.2]-propellane core 5 in 73% yield after three reaction cycles [2].

**Scheme 2:**
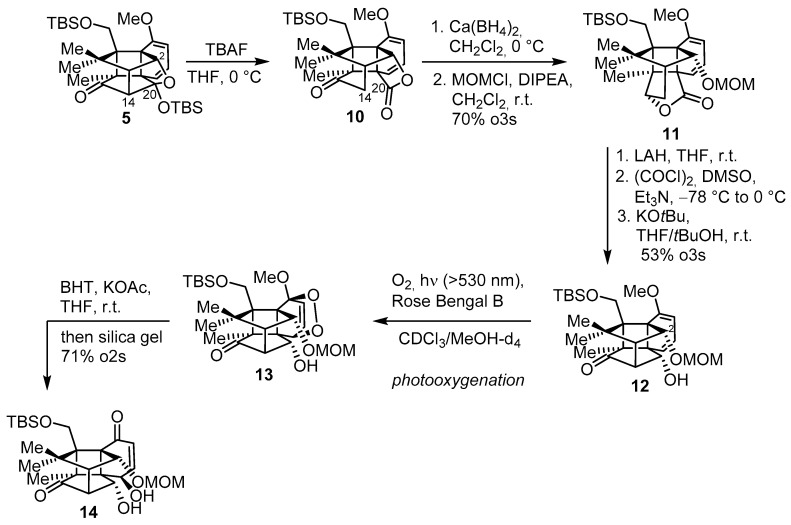
Retro aldol fragmentation–aldol–photooxygenation sequence towards the synthesis of canataxpropellane (**4**) [2]. MOM = methoxymethyl; DIPEA = Hünig’s base; BHT = butylhydroxytoluene.

At this stage, “only” TBS-protected hemiacetal had to be deprotected, but this proved a challenging task, and therefore, an elegant and well-balanced sequence of additional steps had to be established in order to arrive at compound **12**. The treatment of TBS-protected lactol 5 with tetrabutylammonium fluoride led to the undesired retro-aldol fragmentation of the C-14–C-20 bond instead of ketal cleavage. The reformation of the C-14, C-20 C–C-bond was achieved by the reductive lactone opening of 10, Swern oxidation of both the primary and secondary alcohol and a subsequent aldol reaction. Using photooxygenation, they were able to covert the vinylogous enol ether into endoperoxide **13**, which was subsequently converted to enone **14** [2] (Scheme 2).

**Scheme 3:**
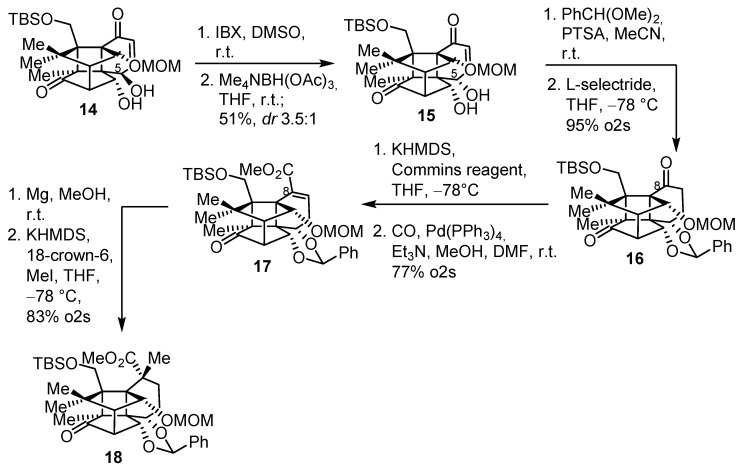
Inversion at C-5 and carbonylation of C-8 towards the synthesis of canataxpropellane (**4**) [2]. MOM = methoxymethyl; IBX = 2-iodoxybenzoic acid; PTSA = *p*-toluenesulfonic acid.

After inversion of alcohol at C-5, acetalization of **15** and 1,4-reduction afforded acetal **16**, which was further carbonylated at C-8 via the corresponding enol triflate (Scheme 3). A magnesium-effected 1,4-reduction followed by methylation led to desired ester **18** [2].

**Scheme 4:**
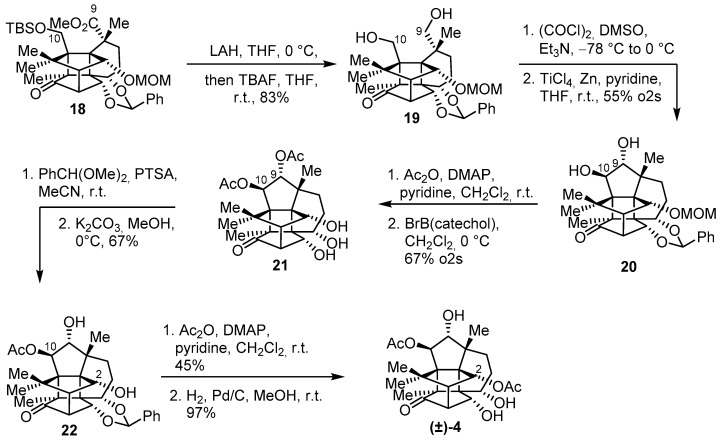
Endgame of Gaich’s canataxpropellane (**4**) synthesis: *trans*-selective pinacol coupling and selective acylation of secondary alcohols [2]. MOM = methoxymethyl; PTSA = *p*-toluenesulfonic acid; DMAP = *N*,*N*-dimethylamino pyridine.

For the construction of the last C–C-bond between C-9 and C-10, a trans-selective titanium-catalyzed pinacol coupling furnished the desired *trans*-diol **20**. Acetylation of the alcohols at C-9 and C-10 and deprotection of the methoxymethyl (MOM)-ether provided **21** as a mixture with its acetal. Via reacetalization and hydrolysis of the acetal at C-10, they obtained **22** in 67% yield. In the endgame, Gaich and coworkers acetylated the secondary alcohol at C-2 selectively and cleaved the acetal via hydrogenation conditions [2]. The completion of this total synthesis is a milestone in recent synthetic history and was supported by a recent highlight article in Angewandte Chemie by the Heretsch group [3]. By solving a longtime synthetic challenge, the first total synthesis of canataxpropellane (**4**) gives rise to strategies for further complex targets in natural product synthesis (Scheme 4).

### 2.2. Waihoensene

**Figure 4:**
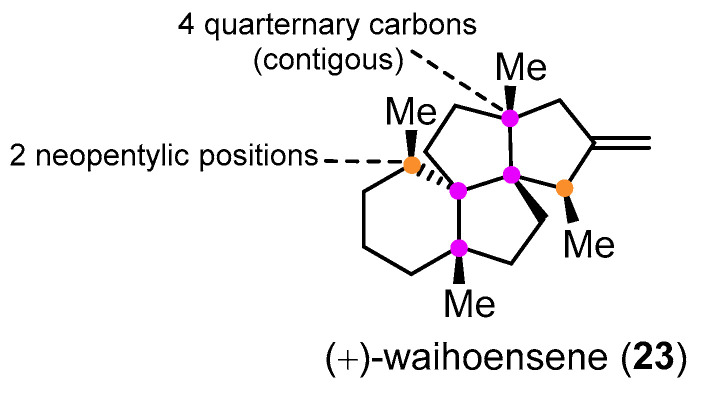
Structure of (+)-waihoensene (**23**).

Waihoensene (**23**) is a secondary metabolite natural product for which synthesis was considered to be particularly challenging. Its structure was published in 1997 by the group of R. T. Weavers as an isolation product from the New Zealand conifer *Podocarpus totara var. waihoensis* [9]. It features four contiguous quaternary stereocenters and two associated chiral neopentylic positions. The combination of chiral centers and condensed carbocyclic ring systems of this laurenene-related diterpene initiated a variety of chemical syntheses, of which three are covered herein (Figure 4).

**Figure 5:**
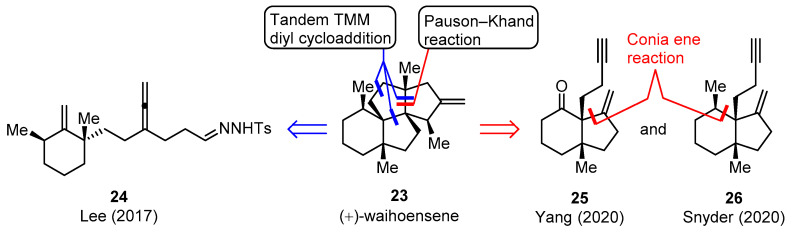
Comparison of the retrosynthetic strategies.

When looking at the total syntheses of waihoensene (**23**) published to date, two different strategies can be noted (Figure 5). The group of Lee made use of a tandem cycloaddition involving a trimethylene-methane diradical (TMM diyl) intermediate, which was established by the same group. This transformation builds up three bonds, and therefore the angular triquinane moiety, in one step. The pivotal sequence of this synthesis is a reaction cascade combining an intramolecular [2 + 3]-dipolar cycloaddition, generation of the TMM diyl intermediate through the loss of nitrogen and a second [2 + 3] cycloaddition [4].

The groups of Yang and Snyder made use of both a Conia-ene reaction to install the bicyclic system with two quaternary centers in place and a Pauson–Khand reaction to assemble the tetracyclic core, setting another quaternary center [5,6].

In 2017, the group of Lee was the first to succeed in the total synthesis of the tetracyclic diterpene (±)-waihoensene (**23**) [4]. In this 18-step synthesis, a tandem cycloaddition proceeding through a trimethylenemethane (TMM) diyl intermediate was employed to build up the angular triquinane moiety of the tetracyclic core.

**Scheme 5:**
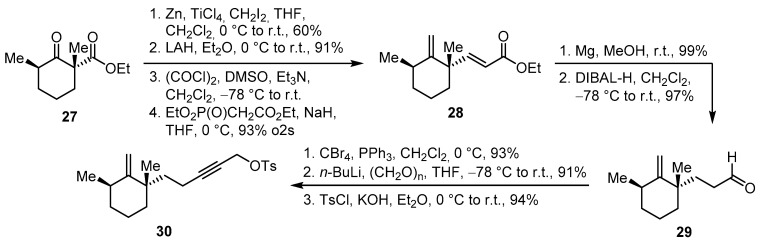
Synthesis of progargylic tosylate **30** from ketoester **27**. Ts = toluenesulfonyl.

In the beginning, ketoester **27**, which was derived from ethyl 2-cyclohexanonecarboxylate in two steps, was transformed into aldehyde **29**. After a Corey–Fuchs reaction and an in situ trapping of the alkynyl anion with formaldehyde, the resulting propargylic alcohol was treated with TsCl to afford tosylate **30 [4]** (Scheme 5).

**Scheme 6:**
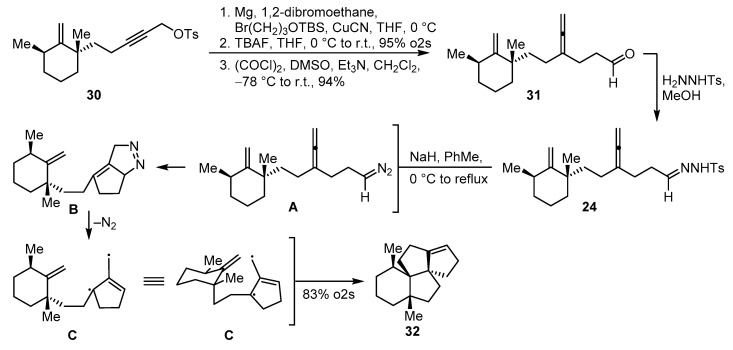
Synthesis of key intermediate **24** and subsequent cycloaddition cascade to tetracycle **32**. Ts = toluenesulfonyl; TBS = *t*-butyldimethylsilyl; TBAF = tetra-*n*-butylammonium fluoride.

Allenyl aldehyde **31** was generated by reacting tosylate **30** with the Grignard reagent Br(CH_2_)_3_OTBS in a copper(I)-catalyzed S_N_2′ reaction, followed by cleavage of the TBS-ether and Swern oxidation of the liberated alcohol. After 12 steps and with allenyl aldehyde **31** in hands, the stage for the key step tandem cycloaddition reaction was set. Subjecting tosylhydrazone **24** to sodium hydride led to the formation of the corresponding diazo compound **A**, which would initiate the first [3 + 2]-cycloaddition, giving **B** as an intermediate species. The loss of nitrogen then generated the TMM diyl intermediate **D**, which underwent the second [3 + 2]-cycloaddition to build up **32** as the major product alongside other isomeric products (Scheme 6). In sum, this transformation not only assembled the angular triquinane moiety but also set two all-carbon quaternary stereocenters [4].

**Scheme 7:**
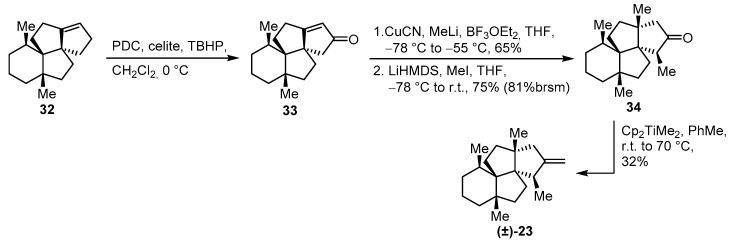
Endgame of Lee’s total synthesis of (±)-waihoensene (**23**). PDC = pyridinium dichromate; TBHP = *t*-butyl hydroperoxide.

For the completion of the synthesis, the introduction of two methyl groups and the *exo*-methylene group was required. For this, enone **33**, which was derived from tetracycle **32**, was subjected to the addition of a higher-order cuprate to the less hindered α-face. The last stereocenter was constructed by regioselective enolate formation using LiHMDS and subsequent methylation of this α-position, yielding tetracyclic ketone **34** as a single isomer. Eventually, the *exo*-methylene group was installed via Petasis olefination to complete the first total synthesis of (±)-waihoensene (**23**) [4] (Scheme 7).

In 2020, the first asymmetric synthesis of (+)-waihoensene (**23**) was completed by Yang and coworkers in 15 steps and 3.8% overall yield [5]. Here, well-considered transformations like a Conia-ene-type reaction, a Pauson–Khand reaction, and an intramolecular hydrogen atom transfer (HAT) were employed as key steps on route to (+)-waihoensene (**23**).

**Scheme 8:**
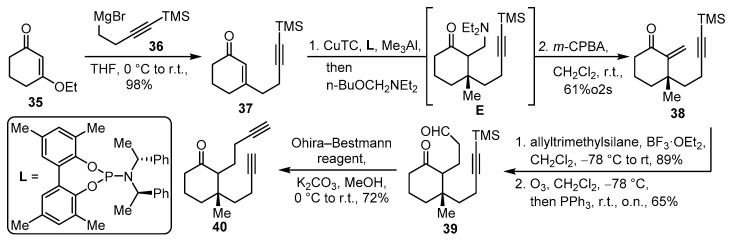
Synthesis of Conia-ene reaction precursor diyne **33**. TMS = trimethylsilyl; CuTC = Copper(I) thiophene-2-carboxylate.

Starting from vinylogous ester **35**, a sequence containing Stork–Danheiser transposition, asymmetric conjugate addition, Sakurai allylation and Ohira–Bestmann homologation provided diyne **40** in only six steps as the precursor for the Conia-ene reaction [5] (Scheme 8).

**Scheme 9:**
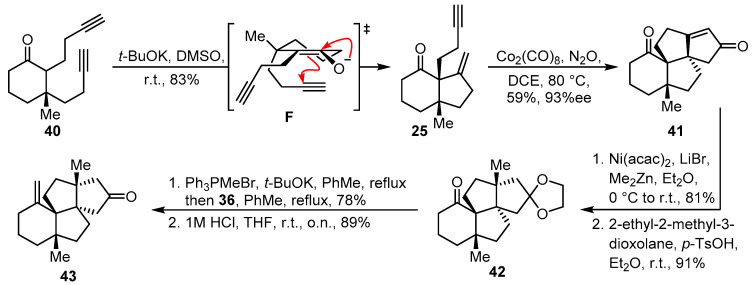
Synthesis of tetracycle **43**. DCE = dichloroethane; Ni(acac)_2_ = bis(2,4-pentanediono)nickel.

Exposing diyne **40** to catalytic amounts of *t*-BuOK initiated a diastereoselective Conia-ene-type cyclization, affording enyne **34** as a single diastereomer. At this stage, two of the four contiguous all-carbon quaternary stereocenters are set. With Co(CO)_8_ and nitrous oxide, enyne **25** underwent a Pauson–Khand reaction to afford the corresponding cyclopentenone **41**, thus having assembled the tetracyclic framework containing the angular triquinane. To generate the only remaining all-carbon quaternary stereocenter, a Ni-catalyzed diastereoselective conjugate addition using Ni(acac)_2_ and Me_2_Zn was employed. Next, a Wittig reaction of mono-ketal **42** and subsequent treatment with hydrochloric acid gave tetracycle **43** with all four all-carbon quaternary centers in place [5] (Scheme 9).

**Scheme 10:**
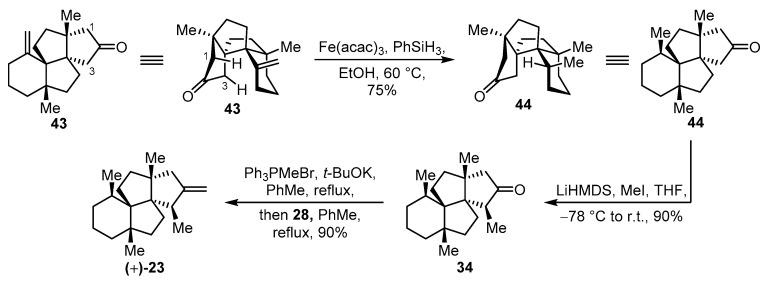
Endgame of Zhang’s total synthesis of (+)-waihoensene (**23**). Fe(acac)_3_ = Tris(acetylacetonato) iron(III).

Since diastereoselective hydrogenation of the double bond in tetracyclus **43** was not feasible and would mostly give the undesired stereochemistry at the methyl-bearing stereocenter, hydrogen atom transfer (HAT) reactions were considered. Generating a carbon radical by employing Baran’s conditions [10] initiated both the intramolecular [1,4]- and [1,5]-HAT of the hydrogen atoms at C-3 and C-9, leading to product **44** with the desired stereochemistry. Eventually, α-methylation, as performed by Lee and coworkers, and Wittig olefination of the ketone completed the synthesis of (+)-waihoensene (**23**) [5] (Scheme 10).

**Scheme 11:**
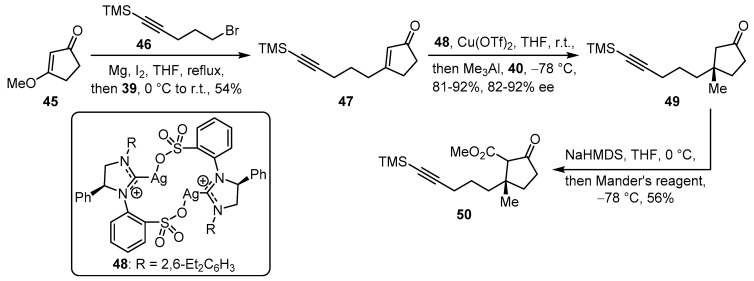
Synthesis of ketoester **50**. Tf = trifluoromethanesulfonate.

Shortly after the publication of Yang’s total synthesis, the group of Scott Snyder published the third total synthesis of (+)-waihoensene (**23**), giving the natural product in 17 steps [6]. Here, an approach similar to Yang’s, including a Conia-ene reaction as well as a Pauson–Khand reaction, was chosen [6] (Scheme 11).

**Scheme 12:**
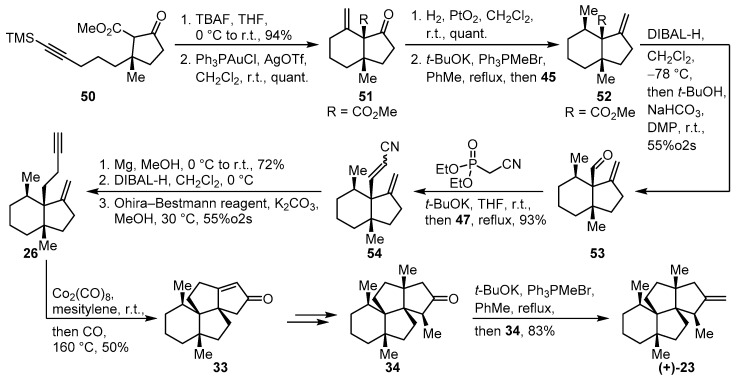
Snyder’s synthesis of common intermediate **33** and transformation to (+)-waihoensene (**23**). TBAF = tetra-*n*-butylammonium fluoride; Tf = trifluoromethanesulfonate.

The synthesis commenced with a Stork–Danheiser transposition followed by an asymmetric conjugate methyl addition, as described by Hoveyda [11,12], thereby setting the quaternary center to direct the formation of the following three. Deprotonation of cyclopentanone **49** and treatment with Mander’s reagent gave a 1.8:1 mixture of **50** and its regioisomer, which itself could be subjected to Krapcho decarboxylation conditions, giving back cyclopentanone **49** [6] (Scheme 12).

After silyl group removal, a Conia-ene reaction was employed to build up the second ring and therefore the second quaternary center, giving bicyclic ketone **51**. With the bicyclic system in place, facially selective reduction of the double bond from the concave face by hydrogenation with PtO_2_ (3.2:1 *dr*) set the desired methyl group. Similar diastereoselectivity was observed when using Shenvi’s HAT-type reduction [13], which also coincides with the results of the Yang group. After Wittig olefination, the ester was transformed to the corresponding aldehyde **46** using a one-pot sequence that was developed by the group of Snyder [14]. A sequence consisting of homologation steps afforded enyne **26** as the precursor for the sought-after Pauson–Khand reaction. Subjecting enyne **26** to Co_2_(CO)_8_ at 160 °C under a CO atmosphere readily delivered tetracyclic enone **33** with all quaternary centers in place [6].

At this point, tetracyclic enone **33** is the key intermediate of Lee’s total synthesis, and the following known steps were carried out to give tetracyclic ketone **34**. Eventually, the synthesis of (+)-waihoensene (**23**) was completed by making use of the earlier applied Wittig protocol, by which the final step was optimized in yield [6].

### 2.3. Wang’s Synthesis of (±)-11-O-Debenzoyltashironin

The structure of 11-*O*-debenzoyltashironin (**55**) was published in 2001 by Fukuyama and coworkers, who isolated the sesquiterpene from the fruits of *Illicium merrillianum* [15].

**Figure 6:**
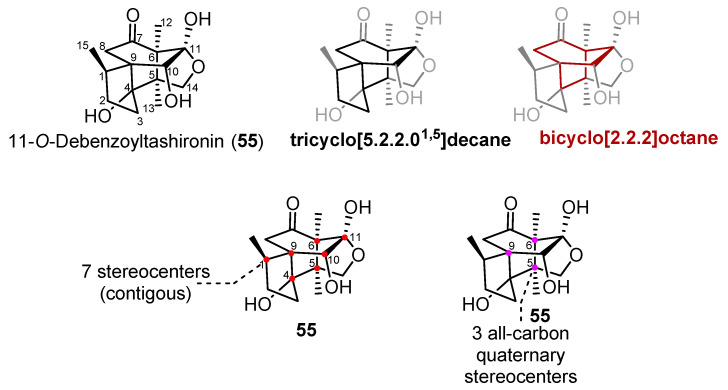
Rings systems and stereocenters of 11-*O*-debenzoyltashironin (**55**).

Debenzoyltashironin **55** possesses a tricyclo[5.2.2.0 ^1,5^]decane carbon framework, which contains a bicyclic [2.2.2] substructure as a very distinct structural motif. It thereby contains seven contiguous stereogenic centers with three all-carbon quaternary centers (C-5, C-6, C-9; Figure 6).

**Scheme 13:**
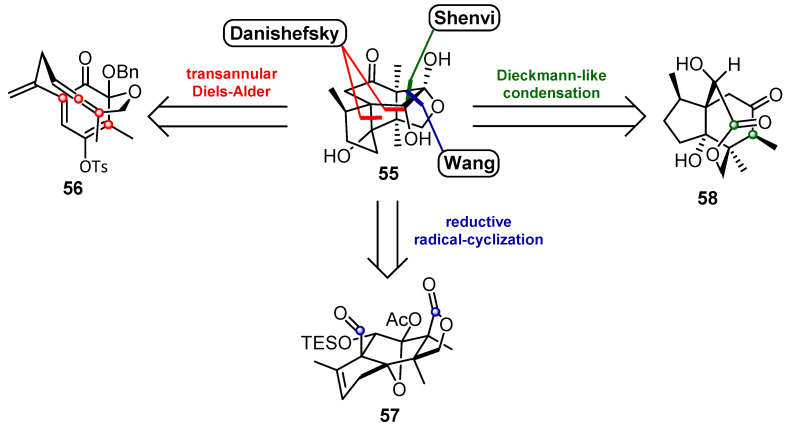
Comparison of key strategies and intermediates in the construction of the tashironin backbone.

11-*O*-Debenzoyltashironin was first synthesized by Danishefsky and coworkers, and it is an exhibition piece for the utility of the dearomatization/Diels–Alder sequence in the construction of bridged core structures. The key two-step sequence thereby set up the entire carbon backbone of the molecule, featuring four rings and three quaternary centers (**56**, Scheme 13) [16]. Although it is very appealing in terms of synthetic elegance, the Danishefsky synthesis was surpassed in efficiency when Shenvi and coworkers published their asymmetric 10-step synthesis 10 years later. Their approach was based on the central intermediate of Shenvi’s jiadifenolide synthesis [17], which was further modified towards ketone **58** to afford the natural product via Dieckmann cyclization (Scheme 13) [18].

In their recent efforts to access the bridged ring systems of *Illicium*-derived sesquiterpenes through reductive radical cyclizations, Wang and coworkers reported the successful synthesis of 11-*O*-debenzoyltashironin through a new retrosynthetic disconnection, applying a pinacol coupling between the aldehyde and lactone of the precursor (**57**) as a key step (Scheme 13) [7].

**Scheme 14:**
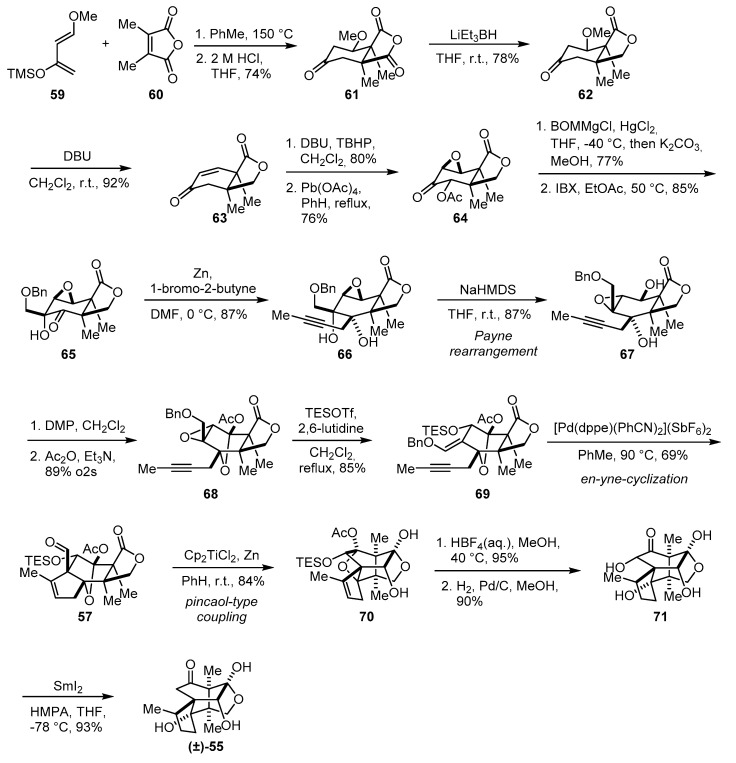
Wang’s total synthesis of (±)-11-*O*-debenzoyltashironin (**55**) [14]. BOM = benzyloxymethyl; IBX = 2-iodoxybenzoic acid; DMP = Dess–Martin periodinane; dppe = 1,2-bis(diphenylphosphino)ethane.

Wang and coworkers started from Fukuyama’s modification of Danishefsky’s enone (**63**) [19,20] and introduced modified Scheffer-Weitz conditions followed by α-oxidation using lead tetraacetate to produce the equatorial epoxy acetate **64** (Scheme 14). Introduction of the side chain was performed via mercury(II) chloride-catalyzed Grignard addition. Subsequent saponification and IBX oxidation furnished ketone **65**. Further addition of a propargylic zinc organyl established the foundation for base-induced Payne rearrangement of the tertiary alcohol to shift the epoxide and liberate secondary alcohol **67**. Oxidation using Dess–Martin periodinane and treatment with acetic anhydride established acetylated mixed ketal **68**. Epoxide opening, simultaneous silyl protection and introduction of *O*-benzyl enolether **69** were effected by standard TES-protection conditions [7].

Pentacycle **69** was closed via a palladium-catalyzed 5-endo en-yne-cylization, which, as a side effect, simultaneously cleaved benzyl ether to obtain aldehyde **57**. The last desired C-C bond was formed by a transannular Cp_2_Ti(III)Cl-mediated pinacol-type coupling of an aldehyde and a lactone carbonyl to free lactol **70**. Acetic TES-deprotection and hydrogenation of the trisubstituted double bond furnished α-hydroxy ketone **71**. The endgame was a samarium(II)iodide-mediated α-deoxygenation of hydroxyketone **71** to finish the synthesis. Starting from Danishefky’s enone **63** [19,20], Wang and coworkers synthesized (±)-11-*O*-debenzoyltashironin (**55**) in a sequence of 14 steps [7].

### 2.4. Total Synthesis of (−)-Illisimonin A by Rychnovsky and Burns

**Figure 7:**
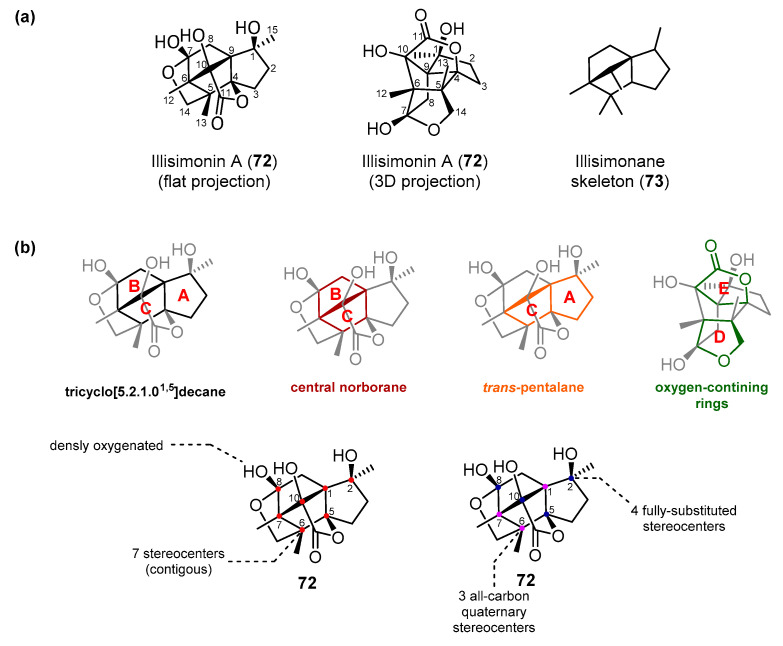
(**a**) Structure of illisimonin A (**72**) in flat and three-dimensional projection, and representation of the illisimonane carbon skeleton (**73**); (**b**) ring systems and stereogenic centers of illisimonin A (**72**).

The structure of illisimonin A (**72**) was published by Ma and coworkers in 2017, who isolated the pentacyclic sesquiterpene from the fruits of the plant *Illicium simonsii* [21]. Its discovery marked a novel extension of the structural scope known to *Illicium-*derived sesquiterpenes with the natural product featuring the unpreceded illisimonane backbone (**73**) (Figure 7a), which combines a norborane (rings B and C) and *trans-*pentalane substructure (rings A and C), making it significantly strained. The backbone is further bridged by a *γ*-lactone and *γ*-lactol ring (rings D and E), which contribute to the cage-like structure of the molecule (Figure 7b).

Illisimonin A (**72**) harbors seven contiguous stereocenters with three all-carbon quaternary centers holding together the molecule’s carbon backbone (C-5, C-6 and C-9) and four additional fully substituted stereocenters (C-2, C-5, C-8, C-10) originating from its dense oxidation pattern (Figure 7b).

In mid-2019, less than two years after the reported isolation and structure elucidation [18], Rychnovsky and Burns published the first total synthesis of the pentacyclic sesquiterpene illisimonin A (**72**) [8].

**Scheme 15:**
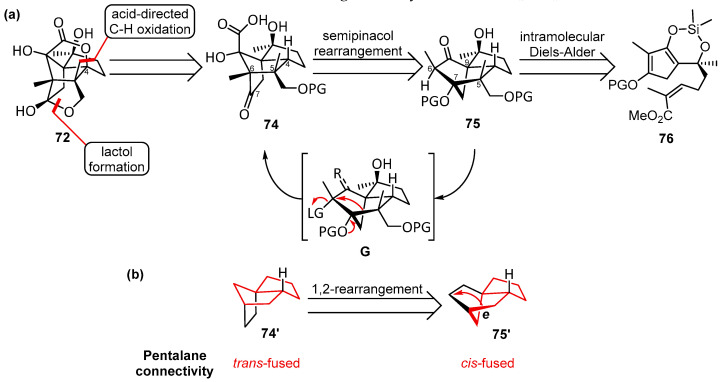
Rychnovsky and Burns’ retrosynthesis of illisimonin A (**72**). (**a**) The three key transformations for the construction of the pentacyclic structure of illisimonin A: intramolecular Diels–Alder, semipinacol rearrangement and carboxyl-directed C-H oxidation. PG = protecting group; LG = leaving group. (**b**) Analysis of ring connectivity and strain as a basis for the rearrangement approach.

Retrosynthetically, Rychnovsky and Burns’ first key transformation is the disconnection of the central gamma-lactone, which they planned to form via a carboxylic acid-directed C-H oxidation of CH-4. They stated that the “gamble” [8] to implement this transformation at such a late stage in the synthesis was motivated by Synder’s scaparvin B-D syntheses and Maimone’s pseudoanisatin synthesis in which a comparably congested methine group was selectively oxidized using White and Chen’s method [8,22,23]. Disconnection of the ketol ring to the corresponding ketone and protected alcohol reduced the natural product to precursor **74**, featuring its complete carbon backbone (Scheme 15) [8].

In their thorough analysis of ring connectivity, Rychnovsky and Burns reported that the tricyclo[5.2.1.0 ^1,5^]decane carbon-framework, as present in the natural product, features a *trans*-fused pentalane substructure (**74′**, Scheme 15b), which is thought to burden the ring system with a significant amount of strain. In contrast, the stereoisomeric tricyclodecane **75′** containing a *cis*-fused pentalane substructure has been shown to be significantly less strained [8,24].

The beauty of Rychnovsky and Burns’ approach to the illisimonane skeleton lies within their realization that the stereoisomeric carbon frameworks **74**′ and **75**′ can be interconverted by 1,2-rearrangement of one of the norborane’s exendo bonds (*e*, Figure 1): Corey described this as *exo-/endo-*substituent interconversion in norborane systems [24]. In application to the synthesis of illisimonin A, the strained backbone of **67** was planned to be accessed from the less strained precursor **75** via semipinacol rearrangement of an intermediate like **G**. Construction of tricyclus **75** was envisaged via an intramolecular Diels–Alder reaction between the cyclopentadienyl moiety and tethered dienophile of **76**, for which calculations of transition-state energies had predicted the desired *exo*-selectivity [8].

**Scheme 16:**
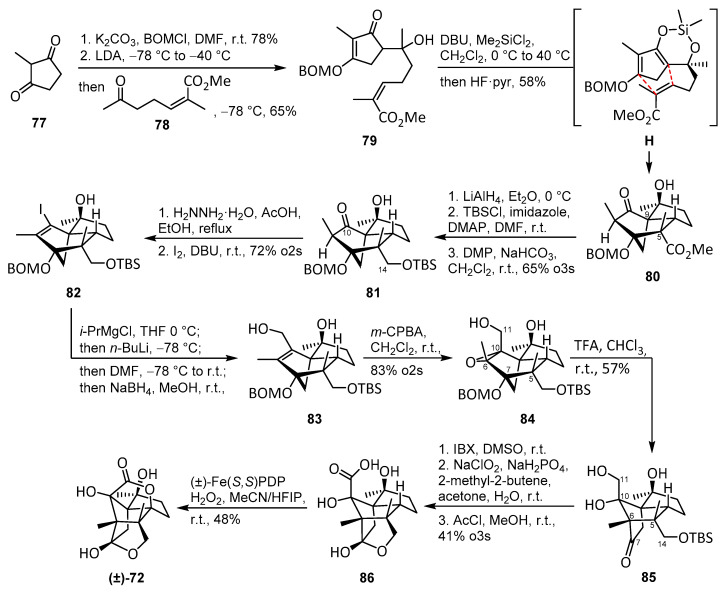
Rychnovsky and Burns’ synthesis of (±)-illisimonin (**72**). BOM = benzyloxymethyl; DMF = *N*,*N*-dimethylformamide; LDA = lithium diisopropylamide; DBU = 1,8-diazabicyclo[5.4.0]undec-7-ene; pyr = pyridine; TBS = *t*-butyldimethylsilyl; DMAP = *N*,*N*-dimethylpyridin-4-amine; DMP = Dess–Martin periodinane; *m*-CPBA = *m*-chloroperoxybenzoic acid; TFA = trifluoroacetic acid; IBX = 2-iodoxybenzoic acid; Fe(*S,S*)PDP = (2*S*,2’*S*) -[*N,N’*-Bis(2-pyridylmethyl]-2,2’-bipyrrolidinebis(acetonitrile)iron(II) hexafluoroantimonate.

Diels–Alder precursor **79** was prepared from cyclopentadienone **77** and underwent the desired cycloaddition to tricyclic key intermediate **80** upon treatment with DBU and dimethyldichlorosilane. The intermediately formed 1,3-dioxa-2-silacyclohexane moiety of **H** thereby excellently templated the facial selectivity of the cycloaddition to stereoselectively build up the two all-carbon quaternary centers at C-5 and C-9 (Scheme 16) [8].

From **80**, an adjustment of the C-14 oxidation state, one-carbon elongation at C-10 to introduce the future lactone carbonyl carbon C-11 and epoxidation were performed to access epoxy alcohol **79** as a suitable substrate for the pivotal semipinacol rearrangement.

Upon treatment with TFA, **84** underwent the envisioned rearrangement, which (a) set up the vicinal quaternary centers at C-5 and C-6 and (b) liberated the appropriate functional groups at C-7 and C-10, as needed in the natural product (**85**, Scheme 16). Rychnovsky and Burns thereby demonstrated once more the utility of the so-called type 3 epoxy alcohol semipinacol rearrangement for setting up 1,3-functional group distances with an interjacent quaternary center [25].

In the endgame of the synthesis, C-11 oxidation and TBS group removal afforded acid **86**, only lacking the *γ-*lactone of the natural product. The final C-H oxidation was achieved using White’s FePDP catalyst in a mixture of acetonitrile and hexafluoroisopropanol to complete the synthesis of (±)-illisimonin A ((±)-**72**) [8]. 

Overall, Rychnovsky and Burns’ synthesis enabled access to racemic illisimonin A (**72**) with an overall yield of 0.5% over 20 steps and a scalable sequence (multigram scale up to **84**) that allowed the synthesis of 100 mg quantities of the natural product [8]. Their solid sequence allowed them to further prepare enantiomerically enriched illisimonin A via advanced-stage racemic resolution to revise its absolute configuration from the enantiomeric structure, originally proposed by Ma and coworkers [21], to the depicted one [8].

In conclusion, new and innovative strategies and methods have been developed over the past several years and applied to complex total syntheses. These achievements have been considered to be the next hurdles to be taken in order to advance organic synthesis to the next level. One can imagine that, with these results, the completion of complex natural products will be achieved more rapidly in the future and might help to provide optimized products for medicinal chemistry.
